# An antibiotic stewardship programme to reduce inappropriate antibiotic prescribing for acute respiratory infections in rural Chinese primary care facilities: study protocol for a clustered randomised controlled trial

**DOI:** 10.1186/s13063-020-04303-4

**Published:** 2020-05-12

**Authors:** Chao Zhuo, Xiaolin Wei, Zhitong Zhang, Joseph Paul Hicks, Jinkun Zheng, Zhixu Chen, Victoria Haldane, John Walley, Yubao Guan, Hongyan Xu, Nanshan Zhong

**Affiliations:** 1 National Center for Respiratory Diseases, State Key Laboratory of Respiratory Disease, Guangzhou Institute of Respiratory Health, Laboratory of Guangdong-Hong Kong -Macao Great Bay, Guangzhou Medical University, 151 Yanjiang Xi Rd, Guangzhou City, 510120 Guangdong China; 2 Dalla Lana School of Public Health, University of Toronto, Toronto, ON Canada; 3 Nuffield Centre for International Health and Development, University of Leeds, Leeds, UK; 4 Yuebei People’s Hospital, Shaoguan, Guangdong China

**Keywords:** Antibiotic, Stewardship, Primary care, Smart phone app, Cluster-randomised controlled trial, Acute respiratory infections

## Abstract

**Background:**

Inappropriate prescribing of antibiotics for acute respiratory infections at the primary care level represents the major source of antibiotic misuse in healthcare, and is a major driver for antimicrobial resistance worldwide. In this study we will develop, pilot and evaluate the effectiveness of a comprehensive antibiotic stewardship programme in China’s primary care hospitals to reduce inappropriate prescribing of antibiotics for acute respiratory infections among all ages.

**Methods:**

We will use a parallel-group, cluster-randomised, controlled, superiority trial with blinded outcome evaluation but unblinded treatment (providers and patients). We will randomise 34 primary care hospitals from two counties within Guangdong province into the intervention and control arm (1:1 overall ratio) stratified by county (8:9 within-county ratio). In the control arm, antibiotic prescribing and management will continue through usual care. In the intervention arm, we will implement an antibiotic stewardship programme targeting family physicians and patients/caregivers. The family physician components include: (1) training using new operational guidelines, (2) improved management and peer-review of antibiotic prescribing, (3) improved electronic medical records and smart phone app facilitation. The patient/caregiver component involves patient education via family physicians, leaflets and videos. The primary outcome is the proportion of prescriptions for acute respiratory infections (excluding pneumonia) that contain any antibiotic(s). Secondary outcomes will address how frequently specific classes of antibiotics are prescribed, how frequently key non-antibiotic alternatives are prescribed and the costs of consultations. We will conduct a qualitative process evaluation to explore operational questions regarding acceptability, cultural appropriateness and burden of technology use, as well as a cost-effectiveness analysis and a long-term benefit evaluation. The duration of the intervention will be 12 months, with another 24 months’ post-trial long-term follow-up.

**Discussion:**

Our study is one of the first trials to evaluate the effect of an antibiotic stewardship programme in primary care settings in a low- or middle-income country (LMIC). All interventional activities will be designed to be embedded into routine primary care with strong local ownership. Through the trial we intend to impact on clinical practice and national policy in antibiotic prescription for primary care facilities in rural China and other LMICs.

**Trial registration:**

ISRCTN, ID: ISRCTN96892547. Registered on 18 August 2019.

## Background

Inappropriate use of antibiotics is a major contributor to the global public health challenge of antimicrobial resistance, and has been widely documented in the world, especially in low- and middle-income countries (LMICs) [1, 2]. Rising levels of antimicrobial-resistant infections may devastate our health system, costing many lives and using valuable resources [3, 4]. Acute respiratory infections (ARIs) are commonly seen in primary care consultations, with upper respiratory tract infections (URTIs) accounting for the largest source of inappropriate antibiotic use [5, 6]. This is because most URTIs are viral and self-limiting, and the administration of antibiotics is not clinically effective at shortening duration or symptom severity [7]. Similarly, among lower respiratory tract infections (LRTIs), acute bronchitis is commonly viral, but up to 80% of acute bronchitis patients still receive antibiotics [8]. Bacterial respiratory infections in primary care include acute otitis media, acute sinusitis and group A streptococcal pharyngitis and pneumonia, where narrow-spectrum antibiotics should be recommended as the first choice according to US [9] and UK guidelines [10]. However, broad-spectrum antibiotics, such as macrolides and fluoroquinolones, are often given which are more likely to generate resistance [11–13].

In China, inappropriate use of antibiotics is rampant and has raised national attention. This is particularly challenging in rural areas, as rural health workers have less education/training and may inappropriately prescribe more frequently [14]. A cross-sectional study in 10 provinces in Western China showed that antibiotics accounted for over half of the total prescriptions issued, and mostly for ARIs [15]. China has issued policies to regulate the use of antibiotics over the past 10 years. In 2012, the Chinese MoH issued a regulation for antibiotic prescriptions, limiting them to less than 60% of all prescriptions for inpatients and 20% for outpatients [16]. However, no operational details have been given. The policy has not been proven effective at improving appropriate antibiotic prescribing [17]. Additionally, the policy only focussed on large hospitals and neglected primary care facilities [18, 19].

Interventions addressing inappropriate antibiotic prescribing in primary care that only target patients often show no impact, while effective interventions have employed multi-component strategies that improve the knowledge, attitudes and behaviours of both healthcare providers and patients [20–22]. However, most intervention trials have been done in high-income countries [23, 24]. We previously developed an intervention to reduce inappropriate antibiotic prescribing for childhood (2–14 years old) upper respiratory tract infections (URTIs) in rural, public, primary care facilities known as township hospitals. The intervention included education for doctors and caregivers, as well as antibiotic prescribing peer-review meetings for doctors. We evaluated the intervention in a cluster-randomised controlled trial (cRCT) in Guangxi province, China, and it successfully reduced the frequency of antibiotic prescribing for childhood URTIs by 29 percentage points (95% CI − 42 to − 16) [25]. The interventions also demonstrated long-term benefits 12 months after trial completion [26].

The *WeChat* app is widely used in China. It allows multi-purpose messaging which can facilitate real-time communications among doctors, and between patients and doctors. This app has previously been effectively used in mass public health education programmes [27, 28]. In Tibet, we have employed the WeChat app to link with electronic medical records (EMR) and monitoring boxes to support tuberculosis patients in completing their treatment under harsh travel conditions [29]. However, these technologies have not been applied in reducing inappropriate use of antibiotics.

Based on our study in Guangxi, we aim to enhance our existing intervention by establishing an antibiotic stewardship programme in primary care facilities to address inappropriate prescribing of antibiotics for any form of ARI other than pneumonia, in patients of all ages, with the educational package targeting physicians and patients/caregivers. The intervention will incorporate the WeChat app and make use of EMR. Although the intervention is primarily focussed on public primary care facilities (where all data will be collected) we will also include a separate educational component addressing inappropriate prescribing of antibiotics by private village doctors who work in private village clinics. We will then pilot-test the intervention for feasibility and acceptability of the intervention and trial processes. Assuming feasibility and acceptability, we will then use a cRCT to evaluate the intervention’s effectiveness at reducing inappropriate prescribing of antibiotics for RTIs (and improving other outcomes) in outpatients of any age within public primary care facilities, in comparison to usual care (which means existing relevant standard processes only, as our previously developed intervention has only been implemented in the previous study sites).

## Methods

### Study design and setting

We will test the effectiveness of the intervention using a parallel, two-arm, cluster-randomised, controlled, superiority trial. We will run the trial in township hospitals of two rural counties in Shaoguan city, Guangdong province: Lechang and Nanxiong county. Shaoguan is in the north of Guangdong and remains one of the poorest areas in the province. In rural China primary care is provided by public township hospitals and private village clinics. Each township hospital covers 50,000 to 100,000 people and typically has less than 100 beds and 20–40 family physicians. *Family physicians* are medical doctors who receive formal medical training for either 3 or 5 years to practice acute care, and are employed by the township hospital. Village clinics are run by one or two paramedics (called *village doctors*), who receive limited medical training equivalent to a high-school level, and are guided by the township hospital. Village doctors are self-employed, primarily relying on three sources of income: the consultation fees paid by the rural health insurance, the Government’s public health package for preventative care, and income from private practice which is not properly documented.

Since 2013, the Chinese Government has implemented a ‘zero mark-up policy’, which prevents township hospitals and village clinics from earning profits by prescribing or dispensing medicines on the *Essential Medicines List*. Only medicines on the *Essential Medicines List*, issued by the national and provincial governments, can be prescribed in township hospitals and village clinics. These medicines are purchased through an open bidding platform run by the Provincial Government. Village clinics have access to a much lower variety of medicines compared to township hospitals. Formally, village doctors are not allowed to prescribe antibiotics based on their practice regulations. However, in our previous work in Guangxi we found that some village doctors did actually prescribe antibiotics for common colds, and that villagers often visited village clinics for convenience but frequently got the wrong advice, which is likely to drive demand for antibiotics when they seek care for themselves or their children in township hospitals [30]. For these reasons, village doctors need to be targeted by the invention as well.

### Eligibility criteria

#### Clusters

We define eligible clusters as all willing township hospitals, and family physicians in those township hospitals, that have functional and extractable EMR, along with their associated village clinics, and village doctors in those village clinics, from within the two selected counties of Shaoguan. However, the two township hospitals and their associated village clinics selected for the pilot study (see below) will not be eligible for the trial. Preliminary work suggests that all township hospitals and their associated village clinics within the two counties are eligible.

#### Patients

Our trial targets township hospitals, which provide the majority of primary care in rural China. Thus, we will collect all outcome data from eligible patients’ prescriptions issued within participating township hospitals. Similar to our previous trial setting, all outpatient consultations in China result in a prescription due to the request of health insurance scheme for documenting the consultation and the routine practice to give patients medications/herbs after the consultation. We will not collect prescriptions from village doctors because there is not sufficient documentation (such as prescriptions) available in private village clinics. We define eligible patients as outpatients aged between 0 and 75 years who receive a primary diagnosis of an ARI and receive a prescription following their consultation with a family physician in a township hospital. This includes a diagnosis of any URTIs according to the *International Classification of Disease, version 10* (ICD-10), and acute bronchitis as an uncomplicated acute lower respiratory tract infection (LRTI) (Table 1). However, patients and their prescriptions will not be eligible for inclusion in the trial if they are high-risk patients diagnosed with either: (1) non-ARIs, (2) pneumonia, as it is severe and clinically challenging to group with other uncomplicated ARIs (but we will extend the invention in the future to include pneumonia), (3) chronic conditions including asthma, chronic obstructive pulmonary disease, non-infective or non-acute disorders (e.g. cystic fibrosis, pulmonary embolus, heart failure, oesophageal reflux and/or allergies), non-respiratory infections (e.g. cutaneous infections, urinary tract infections, trauma-related infections, bacterial enteritis and/or cellulitis/abscess), immunological deficiencies, tuberculosis or any form of cancer.

**Fig. 1:**
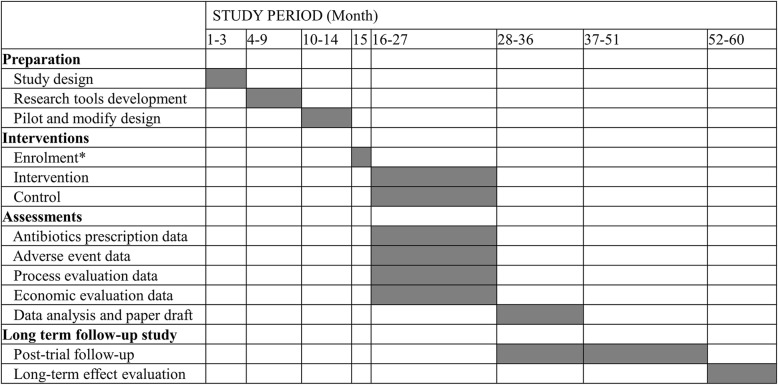
Detailed trial timeline. *Enrolment includes participating township hospitals signing the consent form and relevant healthcare staff being trained

**Table 1:** *International classification of Diseases, version 10* (ICD-10) codes used for acute respiratory infection case definitions

Acute respiratory infections	ICD-10 codes
Acute nasopharyngitis (common cold)	J00
Acute pharyngitis	J02.8, J02.9
Acute tonsillitis	J03.8, J03.9
Acute URTIs of multiple and unspecified sites	J06
Acute bronchitis	J20
Acute sinusitis	J01.0, J01.1, J01.2, J01.4, J01.9
Acute otitis media	H65, H66, H67
Streptococcal pharyngitis	J02.0
Streptococcal tonsillitis	J03.0

*URTIs* upper respiratory tract infections

### Ethical approval

The trial has obtained ethical approval from the Ethics Committee of the First Affiliated Hospital of Guangzhou Medical University, China (2019–53) and the University of Toronto Office of Research Ethics, Toronto, ON, Canada (38265).

### Processes

#### Control arm

In control-arm clusters we will not make any changes or provide any input of any sort. Township hospital and village clinic providers will be allowed to continue treating patients with ARIs according to their existing usual treatment practices and guidelines, and as all data is collected from EMR there will also be no observable impacts for facility staff due to data collection.

#### Intervention

Based on our previous intervention we have developed a comprehensive antibiotic stewardship programme to reduce inappropriate prescribing of antibiotics for ARIs in the general population within rural primary care settings [25]. The intervention targets family physicians and patients in township hospitals, as well as the village doctors associated with township hospitals. The interventional activities are designed to fit within the existing policy requirements around antibiotic prescribing, which are detailed in Table 2. In all our training materials we define appropriate use of antibiotics as: (1) for all URTIs other than bacterial pharyngitis and sinusitis any use of antibiotics is considered inappropriate, and (2) for bacterial pharyngitis, sinusitis, otitis media and bronchitis, amoxicillin or penicillin are the first-line recommended antibiotics and are considered appropriate.

**Table 2:** Intervention package and, where relevant, equivalent control-arm processes

Targeted group	Intervention arm	Control arm
Provider side	1. Improved antibiotic stewardship programme: (a) Each township hospital will establish an antibiotic stewardship working team including the township hospital director and a senior physician. The team will decide local antibiotic policies (b) The antibiotic stewardship team will hold peer-review meetings on antibiotic use monthly throughout the intervention period and embedded within the routine meetings of family physicians. Before each peer-review meeting, the antibiotic stewardship team will hold an internal meeting to prepare prescription reviews. The results will be sent to individual family physicians through WeChat. Each physician will see their performance of appropriate antibiotic prescribing against the hospital average (c) The antibiotic stewardship team will organise monthly peer-review meetings in township hospitals to ensure: (1) clear targets are set for appropriate use, (2) specific feedback is provided to high prescribers on improvement, (3) adjusting assessments for individual physicians based on their performance and (4) recording monthly meeting memos (d) The research team will make monthly supervisory visits to intervention township hospitals. The supervisory visits will ensure: (1) adherence to the interventional activities, (2) feedback and an opportunity to answer questions, (3) meeting with the antibiotic stewardship team to strengthen leadership and their commitment to reduce antibiotic prescriptions 2. Operational guidelines to reduce antibiotic prescribing for ARIs will be used. The guidelines will include patient symptom-based diagnostic algorithms, when to/ not to use antibiotics, and when narrow-spectrum antibiotics are preferred. Based on Chinese guidelines, chest x-ray examinations will be recommended when symptoms indicate pneumonia but clinical signs are not severe, or if a patient makes a second visit with signs indicative of pneumonia. Key health education messages during consultations with patients are also included (a) Every family physician in the township hospitals will receive an operational guideline both in printed and app-based form (b) Village doctors will receive a brief operational guide regarding appropriate use of antibiotics and referral 3. Systematic training including a half-day training workshop run by a county-level hospital senior physician and the research team will be given to family physicians and village doctors. This will involve: (a) Lectures, case discussions, role plays and Q&As on appropriate antibiotic prescribing practices for RTIs based on operational guidelines. Every family physician in the township hospitals will receive a training booklet both in printed and WeChat-app form (b) Using the WeChat app to monitor antibiotic use based on monthly reports from the EMR system to facilitate physicians’ discussions (c) Communication skills training when consulting with patients and/or caregivers 4. Improved electronic prescription system in the EMR to help township hospital family physicians make appropriate decisions when treating patients for RTIs, involving: (a) Embedded modules to encourage appropriate antibiotic prescribing practice, such as pops-up of laboratory test checklists and recommended antibiotics to prescribe when prescribing (b) An alarm system will be implemented to alert the family physician about any patient who re-visits any hospital within 14 days or who has been admitted within 1 month for a respiratory infection or sepsis after their index visit. In this scenario, chest x-ray and blood profile examinations will be recommended and recorded	1. Currently most township hospitals have an existing antibiotic stewardship programme. However, the programmes are often not functioning because of specific targets and activities are planned/implemented. Township family physicians do already hold monthly administrative meetings, but no time is spent on antibiotic prescribing peer-review discussions 2. Township hospital family physicians will prescribe antibiotics according to existing national guide on use of antibiotics which are not specifically designed for primary care facilities, and rarely used. Most physicians prescribe based on their group practice and existing knowledge/practices. Any messages given by control-arm family physicians will be at their own discretion based on their existing practice and training 3. Township hospital family physicians receive relevant medical training before they obtain their qualifications, but only a few of them have opportunities to receive continuing training afterwards and these trainings are usually not specifically for respiratory infections or antibiotics use. We will not provide any training to control-arm family physicians 4. Township hospitals already have a similar electronic prescription system in the EMR, but there is no clinical decision module, alarm system or pop-ups for recommended and non-recommended antibiotics. We will not make this available for control-arm hospitals
Consumer side (patients/caregivers)	1. Printed educational material and WeChat-app versions describing appropriate antibiotic use for respiratory infections will be available during the intervention period. These educational materials along with education videos will be accessible for the public in the township hospital public areas 2. Patients will be invited to scan a QR code to link their WeChat account to the township hospital public WeChat account for receiving health education materials, making queries and comments, and viewing suggestions about common queries	1. There are and will be no health education materials on appropriate antibiotic use available in control-arm hospitals 2. Currently, township hospital do not have WeChat public accounts for patients, so we will not implement them in control-arm hospitals

*ARI* acute respiratory infection, *EMR* electronic medical records, *RTIs* respiratory tract infections

### Pilot-testing interventions

Before the trial we will test the feasibility of the intervention strategies and research design using qualitative and quantitative methods. Specifically, we will explore the feasibility and acceptability of the intervention strategies from the perspective of the hospital management staff and family physicians, village doctors and patients/caregivers. We will also explore the feasibility of the study design process, including recruiting family physicians and recording the number of eligible outpatient visits.

#### Key activities

We will purposively select two township hospitals, with one township hospital randomly allocated to the intervention treatment and the other allocated to the control arm. We will also invite private village doctors associated with the intervention site for the initial training. We will test all interventions and study processes in one township hospital, while observing what the usual care is in another. We follow the same informed consent process as outlined in the trial.

#### Pilot evaluation

The pilot study will run for 3 months. For the pilot evaluation we will employ a mixed-methods approach and collect data using in-depth interviews with family physicians in township hospitals and patients, as well as a questionnaire administered to all family physicians after training. The questionnaire methodology is very similar to that used in our process evaluation (see the ‘Process evaluation’ section). The pilot evaluation is guided by the MRC framework for process evaluations [31]. A planning matrix, adapted from our previous trial in Guangxi, can be found in Additional file 1 that illustrates our objectives, intended topics, information required, sources of information and methods for data collection. In the intervention cluster we will conduct in-depth interviews with two family physicians, two village doctors and one township hospital director, and we will conduct one focus group discussion with patients or their caregivers. We plan to do five in-depth interviews with family physicians and one focus group discussion with two to three patients or their caregivers during the pilot study. We will administer the questionnaire to family physicians before and near the end of the pilot study period. We will also perform structured observations to record training and implementation (e.g. consultations) processes during the pilot period.

Our qualitative methods adopt an interpretive description approach and we will employ thematic analysis as described by Braun and Clarke [32, 33]. We will analyse qualitative data as soon as possible after it has been collected. This approach allows for reflexive identification of themes from interviews and observations which can feed into subsequent interviews. For example, if interesting issues emerge they can be followed up in further interviews. As this is a mixed-methods pilot evaluation, our qualitative results will be analysed with our quantitative questionnaire results to provide feasibility and acceptability on both the intervention and control clusters, as well as on feasibility of meeting the sample size (of the prescriptions).

At the end of the pilot study period we will decide whether to continue with the full trial based on the analysis of the planning matrix. The key criteria would be based on: (1) having sufficient levels of recruitment to likely meet the required number of prescriptions for the trial, and (2) the intervention implementation is judged to be feasible: specifically, it must appear feasible to train at least 60% of family physicians; 80% of family physicians who are trained employ the intervention guidelines at the end of the pilot evaluation period.

### Outcomes

We will collect all outcomes and variables from EMR data on all prescriptions issued to eligible outpatients attending trial township hospitals during the 12 months before randomisation (the ‘baseline’ period) and the 12-month trial period. We define all outcomes at the cluster level and will calculate them from the individual-level prescription data for analysis as single summary outcome values (proportions/means) per cluster.

#### Primary outcome

Our primary outcome is the proportion of prescriptions for eligible patients (aged 0–75 years with a diagnosed ARI, excluding pneumonia and other complications – see the ‘Eligibility criteria’ section) that contain one or more antibiotics, which (as per related literature) we refer to as the antibiotic prescription rate (APR). As in previous trials [17, 34] we will use the APR to measure the proportion of unnecessary antibiotic prescribing/use, given that most patients with URTIs and acute bronchitis do not benefit from antibiotics.

#### Secondary outcomes

We will also collect and create the following set of secondary outcomes, which will allow us to evaluate whether the intervention affects the proportion of antibiotic-containing prescriptions where the antibiotic(s) are of a specific class or method of delivery. The following set of indicators is based only on those eligible prescriptions containing at least one antibiotic: (1) the broad-spectrum antibiotic prescription rate: the proportion that contain one or more broad-spectrum antibiotics (we define broad-spectrum antibiotics as those antibiotics that act on the two major bacterial groups gram-positive and gram-negative, or any antibiotic that acts against a wide range of disease-causing bacteria); (2) the fluoroquinolone prescription rate: the proportion that contain one or more fluoroquinolone antibiotics; (3) the multiple antibiotic prescription rate: the proportion that contain two or more antibiotics of any kind; (4) the intravenously injected antibiotic prescription rate: the proportion that contain any antibiotics delivered by intravenous injection. As the intervention will address appropriate use of antibiotics for bacterial ARIs, we will also measure, taking the total number of antibiotics prescribed per township hospital as the denominator and (5) the proportion that contain any antibiotics in the *Access* group of the *WHO’s 2019 Essential Medicines List* classification [35]. As all these outcomes have denominators that only include eligible prescriptions containing one or more antibiotics, when analysed they will effectively be creating ‘outcome-based subgroups’ that are defined post randomisation [36]. This is a common, but often unrecognised, issue that can introduce bias into the treatment-effect estimates for such outcomes. However, there are no clearly applicable/feasible solutions for our situation [36], and so we will treat the results for these specific outcomes as exploratory.

We will also collect and create the following secondary outcomes based on all eligible prescriptions. In our previous trial, we observed an increased use of Traditional Chinese Medicines, likely as alternatives to antibiotics, and a widespread misuse of glucocorticoids [17]. Thus, we will also measure: (6) the proportion containing any Traditional Chinese Medicines and (7) the proportion containing any glucocorticoids. Lastly, to evaluate if and how the intervention affects the mean cost of prescriptions issued to eligible patients we will also measure: (8) the average cost of a prescription, based on the cost of any medicines and (9) the average cost of a consultation (one per prescription), based on all costs including medicines, tests and the consultation.

#### Patient safety indicator

We will evaluate whether the intervention appears to increase adverse events; for example, because of antibiotics being withheld for appropriate conditions more frequently due to the intervention, by creating and evaluating an indicator of unintended harms. To do this we will use the EMR to track whether any patients subsequently become hospitalised in any hospital in the Shaoguan Prefecture, including its county- and prefectural-level teaching hospitals, due to respiratory infections or sepsis, within 30 days of their index visit to a participating township hospital during the trial period. This will allow us to calculate a cluster-level hospitalisation rate for trial participants as the number of hospitalisations for respiratory infections or sepsis per 100 outpatient consultations. We will compare both patient safety indicators between treatment arms.

### Sample size

Our sample size is calculated based on our primary APR outcome. Previous exploratory work suggested the existing township hospital APR level to be around 80%. We assume that the treatment will reduce the intervention arm APR by more than 15 percentage points during the trial period, based on how effective the previous related trial was [25], which is viewed as the minimum clinically important effect in the context of the extremely high existing APR levels. Based on our previous trial [25] we assume that the intra-cluster correlation coefficient will be 0.14 in the intervention arm and 0.09 in the control arm here, and based on our exploratory work we assume that we will be able to collect at least 500 eligible prescriptions per township hospital during the trial period, which we assume as our fixed cluster size. Based on these assumptions we estimated that we will require 17 clusters per arm to detect a 15-percentage-point or greater absolute reduction in the APR with 80% power using a two-sided hypothesis test with an alpha of 0.05 [37]. Because our previous trial data indicated potentially important county-level differences in baseline and during-trial-period APR levels and in the magnitude of the treatment effect, we also plan to stratify our randomisation by county. Therefore, this sample size also accounted for an unequal allocation ratio within strata, and, specifically given the number of clusters available in each county, it accounted for a within-county intervention-to-control allocation ratio of 8:9, which we chose in favour of the control arm for logistical purposes.

### Recruitment, randomisation and blinding

We will select and seek to recruit all eligible township hospitals and their associated village clinics (i.e. clusters) in the two study counties. There are 17 eligible township hospitals in Lechang county and 17 in Nanxiong county, but we will exclude the two township hospitals from Nanxiong county that will be part of the pilot (see below). We will seek written informed consent from the director of each township hospital on behalf of family physicians in township hospitals and the use of their EMR data. We will also seek written informed consent from all village doctors when participating in the initial training session. We will collect de-identified patient prescriptions from routine EMR system, so individual patient consent is not necessary. Following recruitment the study statistician (JPH) will randomise all recruited township hospitals at the same time, stratified by county, to the intervention or control arm in an overall 1:1 ratio, but will use an 8:9 intervention-to-control-arm ratio within each county, using a simple custom-written computer program in R [38]. Township hospital family physicians and village doctors in the intervention group will then be invited to attend the training.

As the intervention will be applied at the cluster level all patients visiting township hospitals during the trial period will receive the treatment allocated to their cluster. Due to the nature of the intervention it will not be possible to blind providers or patients/caregivers to treatment allocation. However, we will blind the assessors of the adverse events’ outcome (see below), while all other data will be collected from routine databases, and we will also blind the data analysts.

### Data collection and management

#### Clinical consultations and prescriptions

In township hospitals a prescription is required for each consultation to record the clinical visit to enable patients to receive reimbursements from the rural health insurance scheme. Therefore, although in theory patients may visit a family physician but not obtain a prescription this is rare in practice because patients do not feel that they are ‘being taken care of’ without receiving medication(s) [39]. Also, even when outpatient visit costs are not covered by the health insurance scheme patients still prefer to register their visits because the scheme covers much of their consultation costs [40].

#### Primary and secondary outcomes

Following the baseline and the trial periods we will extract all eligible patient prescription data from the EMR for all township hospitals. The Prefecture Health Information Centre manages all EMR, and will provide encrypted and de-identified electronic prescription data (e.g. names, addresses and health insurance numbers will not be collected). We will then clean the data and enter it into a standard format database to store all outcome and covariate data (including diagnoses, medicines prescribed, medicine and consultation costs, plus patients’ age, sex, insurance status, date of visit, any related treatments, payment details and any re-visit or hospitalisation data). We will also collect relevant covariate data from all township hospitals, including family physicians’ age, sex, experience and qualification level, which we can be link to each prescription via unique prescriber IDs.

To allow us to exclude patient prescriptions from outcomes where the patients have excluding comorbidities, we will also record any comorbidities as per secondary diagnoses shown on prescriptions. We will also check medications listed on prescriptions to link them to possible diagnoses. For example, steroid inhalers with asthma or chronic obstructive pulmonary disease, anti-diabetic medications with diabetes, and anti-hypertensive medications with hypertension. The Prefecture Health Information Centre will use the encrypted personal health insurance numbers in each prescription to identify any long-term comorbidities recorded such as diabetes, hypertension, cardiovascular disease. We will also develop a list of keywords used locally in prescription diagnoses for respiratory infections (which are often symptom based), and will manually screen and code all prescriptions not containing an ICD-10 code of diagnosis.

#### Patient safety indicators

We will collect all inpatient charts from the EMR managed under the Prefectural Health Information Centre for any patients who are hospitalised (in any hospital within the Shaoguan Prefecture, including its county and prefectural-level teaching hospitals) within 30 days of their index visit to a trial township hospital for an ARI or for sepsis. The Prefectural Health Information Centre will provide de-identified patient charts for review after identifying patients as having visited a trial township hospital within 30 days of becoming hospitals, using patients’ encrypted insurance numbers. We will then have these inpatient charts reviewed by a group of three to five physicians (blinded to the township hospital of all patients) who will decide if the hospitalisation is due to one of the following reasons: (1) not providing antibiotics during the index visit; (2) other inappropriate usage of antibiotics, such as prescribing the wrong antibiotics; (3) side-effects from antibiotics or other medications and (4) undetermined. Any patients who are determined to have been hospitalised due to either the first or second of these reasons will be included in the calculation of the adverse events’ outcome.

### Statistical analyses

We will report all results according to the ‘Consolidated Standards of Reporting Trials: Extension for Cluster Trials’ (CONSORT) guidelines [41]. Prior to the end of the trial and before the trial dataset is created we will produce a full statistical analysis plan pre-specifying and detailing all planned analyses. In summary though, in our main results paper we will present appropriate descriptive statistics for all relevant patient, family physician, township hospital and village clinic characteristics, along with appropriate summary statistics and their associated 95% confidence intervals for all outcomes by treatment arm at baseline and during the trial. Then for all outcomes we will produce a main set of estimates of treatment effectiveness using cluster-level methods of analysis suitable for cluster trials. This set of estimates will be used as the primary evidence for determining how effective the treatment appears to be for the primary and all secondary outcomes. There will no interim analyses.

For all outcomes these analyses will follow a two-stage process [42]. For the primary outcome and all secondary proportion-based outcomes we will first fit a multiple logistic regression model to the relevant individual-level binary variable from the prescription data, adjusting for (likely) influential individual- and cluster-level covariates, including the cluster-level value of the outcome during the baseline period (we will fully define all adjustment covariates in the analysis plan). We will then use the model’s residuals to create the cluster-level outcome as covariate-adjusted cluster-specific proportions. We will then estimate the treatment effect based on the covariate-adjusted cluster-specific proportions using a stratified (by county) independent *t* test to compare the cluster-level outcome values in each arm. We will repeat this process for the continuous outcome but using a multiple linear regression model to do the initial covariate adjustment. By estimating covariate-adjusted treatment-effect estimates we will reduce the risk of bias in our results due to imbalances in the cluster-randomisation, and we will increase the precision of the estimates. By ultimately estimating treatment effectiveness using a *t* test, for our cluster-level proportion outcomes we will estimate covariate-adjusted risk differences (i.e. absolute differences in cluster-level outcome proportions), as recommended by CONSORT, and for our cluster-level continuous outcome we will estimate treatment effectiveness as a covariate-adjusted mean difference. We will base our inferences about the effectiveness of the treatment on the outcomes by interpreting the 95% confidence intervals around our outcomes’ treatment-effect estimates along with the associated two-sided *t*-statistic based *p* values. We will adjust all confidence intervals and *p* values for our secondary outcomes for ‘multiple comparisons’ using the Holm method [43, 44].

For our main analyses we will including all clusters originally recruited into the trial as per their original treatment allocations, and all patients who are originally established as being eligible and having received treatment in a trial township hospital during the trial period. We will ensure that all eligible patients who received treatment at a trial township hospital during the trial period are included in our main analyses by dealing with any missing outcome and/or covariate data (used in the two-stage process of adjusting for covariates) using multilevel multiple-imputation methods combined with our cluster-level methods of analysis, if needed [45].

To explore the robustness of our main analysis results we will also do a range of sensitivity analyses. Specifically, we will also produce a set of treatment-effect estimates for all covariate-adjusted cluster-level outcomes but without any multiple imputation of outcome or covariate data (used in the two-stage adjustment process), so that only patient prescriptions that include all outcome and covariate data is included in the analyses (these analyses will also include all clusters, as originally recruited and as per their original treatment allocations, and all patients treated during the trial period with any missing outcome or covariate data imputed). Then we will also produce a final set of treatment-effect estimates for all cluster-level outcomes but without any initial covariate adjustment, but with imputation of any missing outcome data (and again including all clusters, as originally recruited and as per their original treatment allocations, and all patients treated during the trial period with any missing outcome or covariate data imputed). We will adjust the confidence intervals and *p* values from each set of sensitivity analysis results for multiple comparisons as per our main analyses to allow comparison.

Lastly, conditional on obtaining ‘statistically significant’ results for our primary outcome, we will also do a small number of pre-planned subgroup analyses of the primary outcome, which will be fully detailed in the analysis plan. These subgroup analyses will be adjusted for the same range of covariates as the main analyses, and will also include all clusters, as originally recruited and as per their original treatment allocations, and all patients treated during the trial period with any missing outcome or covariate data imputed.

### Process evaluation

We will conduct a mixed-methods, theory-driven process evaluation (PE), guided by the MRC’s 2008 framework [46] and Grant’s framework for process evaluations of cluster-randomised trials of complex interventions [47]. Our PE will be based on the underlying programme theory, namely the social cognitive theory, which views behaviour change and maintenance as a dynamic and reciprocal relationship determined by the person, their environment (their external social context) and their behaviour (their response to stimuli to achieve goals) [48]. Our PE aims to offer an exploration of ‘what worked, for whom and why’ in the implementation of the intervention [49]. Our specific objectives for the process evaluation are: (1) to describe the health system and service delivery context in which the intervention was delivered; (2) to examine recruitment processes, both at the cluster level (township hospital) and the individual level (patient consultations and prescriptions); (3) to report on intervention fidelity, both at the cluster level (training) and the individual level (provider delivery) and (4) to explore the responses to the intervention both at the cluster level (managers and providers) and the individual level (patients). Methods will include document review (e.g. recruitment records, meeting minutes), structured observation of trainings and consultations in the township hospital, questionnaires with township hospital family physicians, and interviews of more than 50 participants including six township hospital directors, 18 township hospital family physicians, 12 village doctors and 18 patients/their caregivers, all distributed between the intervention and control arms at a ratio of 2:1. We will also observe four training sessions in the intervention arm. In addition, we will conduct 12 observations of clinical consultations in the intervention arm and six in the control arm. The sample size of the qualitative study is purposively set according to our previous trial in Guangxi and will be adjusted during the study. Our qualitative methods will be guided by an interpretive description approach, which focusses on developing knowledge to inform clinical practice [32]. We will develop a sampling frame, purposively select participants for inclusion and collect data at 3, 6 and 12 months after the start of the intervention in four clusters in the intervention arm and two clusters in the control arm.

#### Analysis of process evaluation

We will analyse qualitative data from the PE using a framework analysis as described by Gale et al. to identify themes related to our study objectives [50]. This approach allows for the inductive discovery of new themes outside of our framework during analysis. We will transcribe and translate into English for all qualitative data, and do our analyses using NVivo 10 software. We will report our qualitative work following the Consolidated Criteria for Reporting Qualitative Research (COREQ) guidelines [51].

### Costing study

We will conduct an incremental cost-effectiveness analysis along with the trial. The primary outcome in this costing study is the cost per percentage point decrease in the APR (as defined in the ‘Outcomes’ section) in the intervention arm compared to the control arm. We will compare direct costs and outcomes of patients randomised to the intervention arm compared to the usual care arm over the 12-month time horizon of the trial. We will not discount costs and benefits due to the short period of the trial [52]. The perspective adopted for the analysis will be that of the healthcare provider.

#### Data collection

We will develop a questionnaire to collect data on the resources used to deliver the intervention, which we will aim to administer to the directors of all 34 township hospitals between the 9th and 12th months of the trial. We will collect information on: (1) average salaries, in renminbi (RMB), for each level of staff in their hospitals, (2) the duration of consultations, (3) the amount of time spent reviewing prescriptions in preparation for the prescribing peer-review meetings and (4) the frequency and duration of peer-review meetings and the staff involved in each process. We will ensure data quality by using double entry and by checking a random subset of the data.

#### Estimation of resource use and costs

Healthcare resource use includes patient visits to the health facility. We will include the cost of consultations, medications, medication reviews and subsidies. The total cost per patient will be calculated as the sum of the three elements: consultation, medication and medication reviews. We will calculate the total costs to the healthcare provider (township hospital) for the main analysis population (see the ‘Statistical analyses’ section) accounting for clustering and stratification [42].

#### Estimation of implementation costs

Implementation costs represent upfront costs and are estimated and reported separately, and will not be included in the cost-effectiveness analysis. However, policy-makers would need to consider these costs when deciding whether to implement the intervention at scale. We will calculate implementation costs for the software development, including the development of the WeChat app function and the EMR improvement. Then, for each cluster, we will calculate them as the sum of: the cost of a trainer to deliver training on appropriate use of antibiotics when treating acute RTIs, the cost of staff time to attend training, plus the costs of producing one handbook and one set of guidelines (used as information aids in consultations) per training attendee, and the educational videos (displayed in waiting areas) and posters (displayed around the hospital).

### Follow-up study

As with our previous trial [26] we will continue to follow-up both intervention and control-arm clusters for another 24 months after the trial ends to monitor the possible longer-term impact of the intervention. It will be at the discretion of individual township hospitals as to whether to continue the interventional activities after the trial has ended. We will investigate to what extent the township hospitals are willing to continue the interventional activities and the long-term influence on family physicians’ behaviors without any interventional efforts provided by the research team. All analyses will be fully pre-specified in the statistical analysis plan.

### Trial management

Prof. Xiaolin Wei from the University of Toronto and Dr. Chao Zhuo from the Guangzhou Medical University will be the co-guarantees of the trial and will have full access to the trial dataset. We will establish a data management committee (DMC) led by external/independent members to safeguard the safety and privacy of the patients involved, and to ensure that all data is collected according to agreed ethical guidelines, properly stored and only used for research purposes. We will also form a trial steering committee (TSC) led by external members. We will organise teleconference meetings for both the DMC and TSC at the beginning of the study, and then every 6 months until the study completes. The committees may also meet on an ad-hoc basis should the need arise. During meetings we will discuss any protocol modifications. We will also establish a trial management unit (TMU) in Shaoguan, to manage the day-to-day activities of the trial, consisting of three research associates employed locally and two research associates from the University of Toronto.

## Discussion

The study is built upon our recent success in rural Guangxi China [25] where we conducted educational interventions to reduce the irrational use of antibiotics for children with URTIs. Compared with the previous study, this study has a number of strengths.

First, the intervention aims to implement a comprehensive antibiotic stewardship programme in primary care facilities, which is much broader than just educational components. The stewardship programme includes: (1) building up the antibiotic stewardship leadership, (2) developing operational guidelines, (3) conducting appropriate antibiotic prescribing training with family physicians and village doctors, (4) employing EMR and smart phone apps as reminders and prescription review tools and (5) educating patients during and after consultations. Systematic reviews have shown that broader interventions targeting both health providers and patients achieve the largest effect [6, 24]. In this trial, we will target uncomplicated ARIs for all ages which will be more useful to family physicians. We plan to develop another trial to improve rational antibiotic use for pneumonia that will be reported subsequently. To our knowledge, our intervention is the first in the world to conduct a randomised controlled trial for such a comprehensive antibiotic stewardship programme in LMIC primary care settings.

Second, the study has important policy relevance to China and other LMICs. The study is chaired by Prof. Nanshan Zhong, an academician in China who is the Chair of China’s National Antimicrobial Committee. The study aims to develop national guidelines for primary care antibiotic stewardship programmes. Despite the implementation of the National Essential Medicines Policy and Zero-Mark-Up Policy in 2009 and the Administrative Regulation for Clinical Antibiotic Use in 2012 [16], their effects on reducing inappropriate antibiotic prescribing have been limited to large hospitals where the enforcement of appropriate prescribing practices is much better [53]. No change was observed in primary care facilities due to the knowledge gap in primary care providers, loose regulation enforcement and a high demand from patients [17, 18, 54]. Our previous trial in rural Guangxi proved the effectiveness and long-term benefits of educational interventions and prescribing peer-reviews in this setting [25, 26]. Based on it, this study aims to establish a more comprehensive and widely applied stewardship programme that is practical, feasible and effective, and which will then hopefully change national policy and clinical practice to benefit billions of people in China. Given that the overuse of antibiotics is a common practice in all LMICs [55], the trial results should be enlightening to other LMICs to adapt for change.

Third, we will employ new technologies using EMR and smart phone apps for reminders and monthly prescription reviews. In our Guangxi trial, we identified that better pre-forming clusters had a more structured process for antibiotic peer-reviews [26, 30]. Similar studies in the US showed better effects using EMR for doctors to justify their prescriptions [56]. Given the high penetration of smart phones in LMICs, there are great potentials for these new technologies in clinical settings. However, there is limited evidence on using apps in reducing inappropriate use of antibiotics. To our knowledge, this study is the first trial to test the effectiveness of using EMR and a smart phone app for the implementation of antibiotic stewardship in LMICs.

Fourth, we will collect information on hospitalisation due to respiratory infections or sepsis to measure the potential patient safety concerns of our interventions. We could not do so in our previous trial in Guangxi, but the integrated medical system in Shaoguan should enable us to collect all hospitalisation data in the prefecture.

Our study has several limitations. First, according to our experience in Guangxi, the diagnosis in primary care facilities may not adhere to ICD-10 codings. We have developed locally applicable diagnostic codes to match with ICD-10. In addition, we will conduct training in the intervention group to use ICD-10 codes, and to avoid using symptom-based diagnoses. This may cause an imbalance between the two arms. However, our early experience in these sites and our previous trial sites showed that only a very small proportion of instances (< 5%) were symptom-based diagnoses.

Second, patients may instead purchase antibiotics from private pharmacies or village doctors without a prescription. Although the Government has regulated that all pharmacies should not dispense antibiotics without a prescription, we observed from our previous trial that this situation existed but at a limited level. Shaoguan is under the reform of integrating village doctors as semi-public health providers under the management of township hospitals. This may reduce the loopholes in the current practice regarding inappropriate use of antibiotics by village doctors. We are also targeting village doctors in our intervention via training and education.

Third, there may be patients who reside in the catchment area of the nearby control township but seek care in the intervention township hospital. This will be included as a patient visit in the intervention arm because our data will be at the individual prescription level in each township hospital, and not by patient residential townships. This may have some negative impact on any benefits of the intervention, but we do not expect this to be substantive.

Fourth, in measuring patient safety indicators, we will miss any patients who do not visit a hospital due to affordability issues. Universal health coverage has been achieved in Shaoguan Prefecture ensuring that every rural resident is covered. The package has a much lower co-payment for inpatient (10%) compared with outpatient consultations (> 50% and with a very low ceiling). Cases of non-affordability of hospitalisation is extremely rare in Shaoguan nowadays.

### Trial status and dissemination plan

The trial was registered at Current Controlled Trials: ISRCTN96892547 on 15 August 2019 (https://www.isrctn.com/ISRCTN96892547). We have not started recruiting patients and township hospitals at the time of submission. We expect to formally launch the trial in March 2020 or later when the 2019 novel coronavirus outbreak comes under control in China. The pilot work has started under the local ethical approval and has finished by the time of submission. We will disseminate the trial results through research articles and policy briefs. We aim to publish our results in international leading medical journals, and present them at national and international conferences. The total lifespan of the study will be 60 months (Fig. 1).

## Supplementary information

**Additional file 1.**

**Additional file 2.**

## Data Availability

Anonymised patient-level data will be made publicly available for non-commercial use through the repository in Guangzhou Institute of Respiratory Health (http://www.gird.cn/index.php) after publication of major papers. Any requests to view data must be approved by the corresponding author based on China’s Public Data Safety Law.

## Abbreviations

APR: Antibiotic prescription rate
ARI: Acute respiratory infection
DMC: Data management committee
EMR: Electronic medical records
ICD-10: International Classification of Diseases, version 10
LRTI: Lower respiratory tract infection
LMIC: Low- and middle-income country
MRC: Medical Research Council UK
RTIs: Respiratory tract infections
RMB: Renminbi (Chinese currency)
RCT/cRCT: Randomised controlled trial/cluster-randomised controlled trial
TSC: Trial Steering Committee
URTI: Upper respiratory tract infection
US: United States
UK: United Kingdom
